# A catalog of *Xenopus tropicalis* transcription factors and their regional expression in the early gastrula stage embryo

**DOI:** 10.1016/j.ydbio.2016.07.002

**Published:** 2016-07-28

**Authors:** Ira L. Blitz, Kitt D. Paraiso, Ilya Patrushev, William T.Y. Chiu, Ken W.Y. Cho, Michael J. Gilchrist

**Affiliations:** a Department of Developmental and Cell Biology, University of California, Irvine, CA 92697, United States; b The Francis Crick Institute, Mill Hill Laboratory, The Ridgeway Mill Hill, London NW7 1AA, UK

**Keywords:** Transcription factors, Gene regulatory networks, Endoderm, Mesoderm, Ectoderm, Gastrulation

## Abstract

Gene regulatory networks (GRNs) involve highly combinatorial interactions between transcription factors and short sequence motifs in *cis*-regulatory modules of target genes to control cellular phenotypes. The GRNs specifying most cell types are largely unknown and are the subject of wide interest. A catalog of transcription factors is a valuable tool toward obtaining a deeper understanding of the role of these critical effectors in any biological setting. Here we present a comprehensive catalog of the transcription factors for the diploid frog *Xenopus tropicalis*. We identify 1235 genes encoding DNA-binding transcription factors, comparable to the numbers found in typical mammalian species. In detail, the repertoire of *X. tropicalis* transcription factor genes is nearly identical to human and mouse, with the exception of zinc finger family members, and a small number of species/lineage-specific gene duplications and losses relative to the mammalian repertoires. We applied this resource to the identification of transcription factors differentially expressed in the early gastrula stage embryo. We find transcription factor enrichment in Spemann's organizer, the ventral mesoderm, ectoderm and endoderm, and report 218 TFs that show regionalized expression patterns at this stage. Many of these have not been previously reported as expressed in the early embryo, suggesting thus far unappreciated roles for many transcription factors in the GRNs regulating early development. We expect our transcription factor catalog will facilitate myriad studies using *Xenopus* as a model system to understand basic biology and human disease.

## 1. Introduction

Genomes are algorithms for building the adult organism, which in vertebrates is comprised of many different cell types, all descended from the single-cell zygote. Differentiation of cell lineages is gradual but highly orchestrated, controlled by the coordinated expression of transcription factors (TFs) to create the correctly patterned organism with a high degree of reproducibility. Central to this process are gene regulatory networks (GRNs), hardwired in the genomic sequence. GRNs integrate intercellular signaling and physiology to determine which genes are to be expressed at specific times, in specific locations, and within certain bounds of expression levels (Peter and Davidson, 2015). GRNs rely on TFs, acting as combinatorial inputs by binding to short sequence motifs located in *cis*-regulatory modules (CRMs) on target genes. TFs often act through the recruitment of coactivator/corepressor proteins, with chromatin context and covalent base modifications (e.g., DNA methylation) of target genes also acting to restrict TF access to binding sites in CRMs. The complexity of TF-CRM interactions is underscored by studies in *Drosophila* and *Caenorhabditis elegans* suggesting that individual genes may be regulated by as many as 15–18 different TFs (MacArthur et al., 2009; MacNeil et al., 2015). Each cell type is believed to possess a unique TF milieu that specifies cell type functions and dictates developmental potential, or *competence*.

The central role TFs play in early embryogenesis has been intensively studied in the frog *Xenopus*. General outlines of the GRNs involved in germ layer (endoderm, mesoderm and ectoderm) specification and patterning during late blastula to early gastrula stages have been generated (Loose and Patient, 2004; Koide et al., 2005; Sinner et al., 2006; Rankin et al., 2011). Dozens of maternally and zygotically expressed TFs have been implicated in the earliest steps of development, and genome-wide studies examining the interaction between individual TFs and CRMs have recently received attention (Gentsch et al., 2013; Chiu et al., 2014; Gupta et al., 2014; Yasuoka et al., 2014; Wills and Baker, 2015). To understand the design principles of GRNs, it is necessary to have a comprehensive understanding of the TFs available to the system, and this requires cataloguing all the TFs encoded by the genome of the species under study. TF catalogs have been produced for several species and serve as valuable community resources (e.g., Kummerfeld and Teichmann, 2006; Wilson et al., 2008; Fulton et al., 2009; Vaquerizas et al., 2009; Ravasi et al., 2010; Hammonds et al., 2013).

We report here a comprehensive, curated Transcription Factor catalog, encoded by the genome of the diploid frog *X. tropicalis* (Hellsten et al., 2010), combining both manual and systematic identification. The resource contains 1235 TFs belonging to 68 DNA-binding domain (DBD) families. Excluding the large and rapidly evolving C2H2 and H2C2 zinc finger families (310 genes), the frog TF repertoire is highly comparable to that of both human and mouse. We find that 118 (~ 13%) of the human non-C2H2/H2C2 TF set have duplications in the *X. tropicalis* TF repertoire. Finally, we identify > 1000 TFs that are detectably expressed at the early gastrula stage. Of these, 218 showed regionalized gene expression, suggesting that many more TFs function in early development than was previously suspected. These results provide an entry point into gain- and loss-of-function studies to elucidate their roles in specification of tissue progenitor populations. The data presented herein will be valuable not only to *Xenopus* researchers, but also to researchers working on early embryogenesis in other organisms. In addition to identifying genes through their DBDs, we have identified genes by name through synteny and close protein matches in the human TF data, and have made recommendations to the community database, Xenbase (Karpinka et al., 2015), for updates to the current annotation.

## 2. Materials and methods

### 2.1. Identification of Pfam DNA-binding domains

To identify genes whose encoded proteins contain sequence-specific DNA binding domains we used data from the Pfam data-base (Finn et al., 2016; v28.0). We first downloaded all Pfam domains annotated as “DNA binding” in the domain description, combining these with DNA binding domains (DBDs) of well-known transcription factors. Inspection of the lists of human and mouse genes annotated with these domains allowed us to further prune the list, removing non-sequence specific binding proteins, and looking for evidence in the literature that the remaining genes encode sequence-specific DNA binding transcription factors. Our final list is composed of 68 vertebrate sequence-specific DNA binding annotated Pfam domains, which formed the basis for identifying the *Xenopus tropicalis* TF proteins. These are available as Supplementary Table 1.

One caveat to this approach is that some DBD families, although containing known TFs, also contain transcriptional regulators that are currently not thought to be acting through sequence-specific DNA binding domains. Our rule-based strategy required acceptance or rejection of entire families of proteins based on the presence or absence of specific DBDs, and we have not attempted to obtain evidence in support or refutation of all members of each family. One exception to this is the ceramide synthase (CERS) enzymes, which contain degenerate Homeobox domains that lack amino acids critical for DNA binding by this class of DBDs (Levy and Futerman, 2010; Burglin, 2011). We have removed these from the catalog (although they are included in our file of ‘dubious’ TFs, see below). Families included in the catalog, despite some uncertainty over some members, are the ARID, zf_C2HC and HMG box families. The ARID family was included because Arid3a and Arid5b proteins bind DNA in a sequence-specific manner (e.g., Patsialou et al., 2005). The zf_C2HC family: *jarid2, kat7, kdm5, and l3mbtl*, are included on the strength of *myt1* (see Gamsjaeger et al. (2013) and references therein); and the HMG_box *kmt2* genes on the strength of the Sox family and other TFs containing this domain. There may be other families with mixed behavior in our catalog.

### 2.2. Identification of TF genes via Pfam domains

To identify all the TF genes in *X. tropicalis* we first downloaded the complete set of latest transcript gene models (v9) from Xenbase, reconstituting the transcript sequences from the gff file describing their locations on the *X. tropicalis* v.9 genome assembly. These data included gene and transcript IDs, and annotated gene names. In addition we downloaded complete sets of human and mouse proteins from Ensembl (Vilella et al., 2009), database v.83, along with associated Pfam domain data. From these latter we reconstructed the analogous sets of human (1603) and mouse (1489) TF genes.

To provide supporting data for open reading frame (ORF) identification and gene naming we first used BLASTx to search the v9 transcripts against the downloaded human and mouse proteins, using a e-value limit of 10^−8^, soft masking for repeat sequences, and retaining the one best protein match from each mammalian species. ORFs were identified by simple codon counting, identifying the likely translation frame from the BLASTx data.

To identify *X. tropicalis* genes encoding proteins containing DBDs, we translated the transcripts into protein sequences, on the forward strand only, using the EMBOSS Transeq tool (Rice at al. 2000), and searched for DBDs with the Pfam domains on our list using pfam_scan.pl (Mistry et al., 2007). We then combined the output of pfam_scan with the calculated ORF coordinates and frame, retaining Pfam domain locations with given *confidence* value of 1, or an e-value better than 0.01, that would be in the translated protein determined by the ORF. Genes with proteins conforming to these conditions were added to our TF catalog. A complete map of all Pfam DBDs found on our set of transcripts is available as Supplementary Data File 1.

To build on the data generated in previous work on this project with earlier sets of transcripts (v4 and v7, from the respective genome assemblies), and in particular, to retain the previous name assignment annotations (see below), we matched the earlier transcript sequences to the v9 sequences using a combination of reciprocal best BLASTn and exon overlap using data generated by the exonerate program (Slater and Birney, 2005) searching the older transcripts against the v9.0 genome assembly. Whilst most of the older transcripts (1092) mapped to the v9 transcripts, 43 mapped to loci that contained no v9 gene model. We include these by reference to the older gene ID and the v9 locus (see the TF catalog). In addition, there were 100 v9 gene models that did not correspond to an older model or transcript sequence, and these represent additions to the catalog by virtue of updating to the latest transcripts. The older transcripts included a small number of EST contigs (Gilchrist et al., 2004), or ad hoc models from the older genome assemblies corresponding to important known TFs, which would otherwise have been missing. Of these older transcript sequences, there were 13 that did not map to the v9.0 genome assembly, and these are also retained in the catalog, some of which are identified despite the absence of a detectable DBD (those cases we investigated were incomplete transcripts). The complete catalog can be found in Supplementary Table 2. Nucleotide sequences and amino acid translations of all TFs are also available as fasta files in the Supplementary data links.

### 2.3. Name identification of TF genes

To assign gene names to poorly annotated genes, we searched for well-annotated orthologs, or closest homologs, in other species, particularly human. This is because *Xenopus* gene nomenclature largely follows human gene names (see http://www.xenbase.org/gene/static/geneNomenclature.jsp). This was done in two ways: a detailed manual curation, spread over several years followed training at the 2006 *Xenopus tropicalis* annotation jamboree in Walnut Creek, CA, and a more recent, systematic analysis based on nearest human protein match used to support name identification but also to explore frog-specific gene family expansions.

The manual curation consisted of several steps (Blitz, 2012) for rigorous confirmation of each gene's identity. Protein translations from all putative TFs were first used in BLASTp searches to identify best matches in NCBI's non-redundant database with special attention being paid to human best matches. To establish correct gene identity we leveraged the extensive synteny that the *X. tropicalis* genome has with mammalian genomes (Hellsten et al., 2010). Synteny was examined using the web-based viewers provided by Metazome (http://www.metazome.net), Genomicus (http://www.genomicus.biologie.ens.fr/, RRID:SCR_011791) and by employing Xenbase's Gbrowse (http://www.xenbase.org; RRID: SCR_003280). We also performed reciprocal tBLASTn searches between the suspected human ortholog's protein sequence and the *X. tropicalis* genome. Reciprocal best tBLASTn hits ensure that no other known *X. tropicalis* gene has more similarity to the presumptive human ortholog than the gene under investigation (Wall, et al., 2003; Blitz, 2012). Additional TF genes were identified following comparison of known human and mouse TF sets to *X. tropicalis*. We searched by tBLASTn for genes missing from the *X. tropicalis* list to identify these genes, which were often missing their DBDs. This is likely a consequence of either incomplete and/or misassembled gene models or gaps in the genomic sequence.

The systematic analysis utilized the BLASTx data described above, generating the closest known protein match from mouse and human for each transcript sequence. Here we noted where the gene name predicted from the closest match was different from the current annotation, and these feed forward as genes whose annotation could be investigated further by the Xenbase annotators. Gene symbols and names have been deposited with Xenbase, which is a clearinghouse for all *Xenopus* gene information and most TFs annotated by the authors have already been incorporated into Xenbase gene pages. The remaining new gene name assignments are provisional, pending review by the Xenbase annotation team, and oversight by the *Xenopus* gene nomenclature committee where required.

### 2.4. RNA-seq analysis of TF expression

Synchronously developing *Xenopus tropicalis* embryos were obtained by in vitro fertilization using standard methods (Ogino et al., 2006). Using two different clutches of embryos, 30–35 embryos at early gastrula stage 10–10.25 (Nieuwkoop and Faber, 1967) were dissected in groups of 10 embryos in 1 × MMR using an eyebrow hair knife and hair loop. Total dissection time in each case was approximately 2 h with explants incubating in 1 × MMR solution for less than 30 min before being homogenized for RNA isolation. Stage-matched sibling gastrulae were assessed for their stage of development at the time of homogenization and none had developed past stage 10.5. The approximate positions of knife cuts are shown in Fig. 3A. Total RNA was extracted using the acid guandinium isothiocyanate-phenol-chloroform method (Chomczynski and Sacchi, 1987), followed by precipitation with 2.5 M LiCl overnight at 4 °C. The RNA was pelleted and washed twice in 70% EtOH before resuspension in DEPC-treated H_2_O. RNA samples were analyzed using an Agilent Bioanalyzer 2100, which demonstrated that all RNA integrity number scores were between 8.9 and 9.5. PolyA+ selection and library production were performed according to the Illumina Tru-Seq mRNA-seq kit instructions and libraries were ligated using bar-coded adaptors. Libraries were subsequently examined using an Agilent Bioanalyzer 2100, quantitated using the KAPA Biosystems qPCR kit and subjected to multiplexed 50-bp single end sequencing on an Illumina HiSeq2000 instrument. Individual datasets had between 19 and 40 M total reads.

RNA-seq dataset quality was verified using FastQC v.0.11.2 and reads were mapped to the *Xenopus tropicalis* v9.0 genome assembly using RSEM v.1.2.12 using default parameters (Li et al., 2011). Differential expression calling was performed over the v9 gene model set using the EBseq package on R v.3.1.10 (Leng et al., 2013). All fastq files, read counts, and processed TPM values are available at GEO accession number GSE81458.

To analyze TF mRNA behavior across early development, expression profiles derived from our high-resolution RNA-seq time series (Owens et al., 2016) and normalized by maximal expression, were hierarchically clustered using Ward's method (Ward, 1963) with Euclidean distance metrics. The flat clusters were formed based on the condition that the distance between a parent node and any of its child nodes exceeds 5. Enrichment in SSDBD was tested in the flat clusters with the Fisher's exact test (Fisher, 1922). Multiple testing was controlled by FDR computed using Benjamini-Hochberg procedure (Benjamini and Hochberg, 1995), with FDR <0.1 considered significant.

To generate the heatmap of spatial expression patterns, the correlation of the z score of gene expression across different fragments were hierarchically clustered using Ward’s method using Euclidean distance. The ratio of the animal cap TPM and vegetal mass TPM was used to further reorder the dendrogram.

## 3. Results and discussion

### 3.1. Generation of a comprehensive catalog of transcription factor genes for Xenopus tropicalis

Transcription factors (TFs) can be broadly divided into three categories: (1) those that modulate transcription by direct binding to specific DNA sequence motifs through their DNA-binding domains (DBDs); (2) those that act indirectly, through protein-protein interactions with direct DNA binding proteins, or otherwise do not have sequence specificity, including coactivators, corepressors, histones, and chromatin modifying enzymes; and finally (3) factors that comprise the core polymerase complex and its associated machinery required directly for RNA synthesis. We restrict our definition to the first of these categories: those TFs that directly interact with DNA through recognized DBDs.

We first identified 68 Pfam domains that are known to be DBDs or are suggestive of sequence specific TF behavior (see Section 2.1 and Supplementary Table 1). Next, we used these DBDs to search gene models derived from the latest *X. tropicalis* genome assembly (v9.0), and also small numbers of sequences derived from earlier versions of this project that we had shown previously to likely be TFs, which either mapped to loci without a gene model on the v9 assembly, or were not found on the assembly and were deemed *off-assembly* sequences (see Section 2 for more information on this). These included EST clusters (Gilchrist et al., 2004), v7 gene models and specific loci from both the v4 and v7 genome assemblies.

Additional TF genes were discovered by comparing human and mouse TF sets to *X. tropicalis*. These *Xenopus* TFs were not found in our DBD search because their gene models lacked intact DBDs. Curation combined both BLASTp searches to identify genes (via protein matches) of closest similarity to the *X. tropicalis* gene of interest, and also reciprocal tBLASTn searches using the protein sequence of the putative ortholog to search for the closest match in the *X. tropicalis* genome sequence (Wall, et al., 2003; Blitz, 2012). Curation also leveraged the extensive synteny relationships between *Xenopus* and mammals (Hellsten et al., 2010) to assign orthology.

In addition we identified candidate gene names from the closest human protein to each transcript sequence, measured by highest scoring BLASTx matches. Gene names and symbols were applied in accordance with the *Xenopus* gene nomenclature, including alignment with human gene names, and submitted to the *Xenopus* community resource Xenbase (http://www.Xenbase.org/), which acts as a repository for, and curates, *Xenopus* gene information.

We found that the *X. tropicalis* v9 genome encodes a repertoire of 1235 TFs, containing one or more of the 68 DBDs in our list, and these genes form our *X. tropicalis* TF catalog (see Supplementary Table 2 and Supplementary Data Files 2 and 3). From this list of TFs, more than 1030 now have gene name annotations, with more than 519 (> 50%) having been assigned names by either our earlier or our current efforts. Interestingly the gene modeling improvements made in the transition from the v7 assembly to v9 resulted in the loss of numerous models containing potential C2H2 and H2C2 family zinc fingers that we had been previously unable to annotate. These frequently had best BLASTp hits to the same small set of human proteins, notably ZNF84 (32), ZNF850 (25) and ZNF41 (15). Retained in the v9 gene model data we found 310 *X. tropicalis* gene models in v9 that encode only C2H2 and H2C2 zinc fingers (with no other additional DBDs), which we refer to as C2H2/H2C2-only genes. It is clear that the orthology relationships of these genes are complex and multiple. Excluding this class from our total TF count, left us with 925 TFs with at least one DBD that is not a C2H2 or H2C2 zinc finger domain.

Since these zinc finger family genes comprise the most rapidly changing TF family across evolution (Tadepally et al., 2008; Klug, 2010), we were interested to replicate this analysis in human proteins. Of the 1608 human TFs, we found 739 “C2H2/H2C2-only” TF genes, leaving 869 human TFs exploring the wider repertoire of DNA binding domains. Interestingly, this is a smaller number than in frog (925), although not by much. If we add back the unmodeled C2H2/H2C2-only genes to the frog count, we get much closer (1545) to the total TF count in human (1608). Clearly there remains much of interest to be discovered in this enigmatic group of C2H2/H2C2-only genes, or possibly pseudo-genes.

The C2H2/H2C2-only genes that we lost in the transition from v7 to v9 data (as they were no longer modeled on the v9.0 assembly but could be mapped from the v7 gene transcripts onto the v9.0 assembly) we considered to be somewhat unlikely TFs. We include these in the files of 180 dubious transcription factor gene sequences (Supplementary Table 2 and Supplementary Data Files 4 and 5).

### 3.2. One-to-one correspondence between Xenopus tropicalis and human TF repertoires

To better understand the relationships between frog and mammalian TFs, we constructed a table containing all human TFs with the *Xenopus* TFs aligned to their closest human TF (see Section 2), repeating the rows for human genes where they correspond to expanded families of *Xenopus* genes. In addition we included the nearest mouse gene to each frog gene, and also the 1:1 orthologous mouse to human genes (from the Ensembl data, see Section 2). The data are then ordered by the human gene name see Supplementary Table 3). From this it is immediately apparent which human/mouse TFs are not found in frog, although there are still some gaps in the *X. tropicalis* genome assembly, and it is possible that the missing genes have yet to be identified. The frog genes not found in human may be found in the TF catalog where no close human match is reported (29 of them), interestingly these contain predominantly bZIP_1 or THAP domains, compared to 13 THAP domains in the rest of the catalog. We conclude that there is a high one-to-one correspondence between the frog and human despite the ~360 million years since their last common ancestor.

We also found some *Xenopus* TF genes of interest to developmental biologists and a few examples are discussed here. We found a gene encoding *pax6.2* (Xetrov90019190), which is closely related to *pax6* and previously only reported in fishes, subsequently described by others (Ravi et al. (2013); see also Nakayama et al. (2015)). We report the identification of the frog ortholog of fish mix-related *mxtx* genes. While zebrafish *mxtx1* and *mxtx2* orthologs are found in other fish species, orthologs have not been reported in tetrapods. We find a single *mxtx* gene (Xetrov90018120) in *X. tropicalis*, although it appears to be absent from amniotes. We suggest the symbol *mxtx1* for the *Xenopus* gene based on BLASTp and synteny comparisons (data not shown). We identify the homeobox gene *soho1* (Xetrov90029300), first reported in chicken (Deitcher et al., 1994). This gene also appears to be absent in eutherian mammalian genomes but is found in the marsupials (opossum and Tasmanian devil), and in birds, reptiles and fish species. We identify Xetrov90020516 as *arx.2*, a second copy of *arx*. The best BLASTp match is to its ortholog in the spotted gar, *Lepisosteus oculatus*, and it is syntenic to the gar gene and to orthologs in chicken, turtle and opossum. However this gene is also absent from eutherian mammals. Xetrov90001891 encodes an unnamed member of the ZBTB (zinc finger and BTB domain) family of zinc finger proteins that also has a best BLASTp match and is syntenic to its ortholog in gar, but appears to be absent from other vertebrates. Another example is *emx3* (Xetrov90007763), which is present in gar and teleost fishes. Examination of the synteny relationships of these *Xenopus* genes and others not discussed here reveals evolutionary transitions in TF repertoires from the fishes to tetrapods.

### 3.3. Temporal expression dynamics of TF expression across early development

**Fig. 1:**
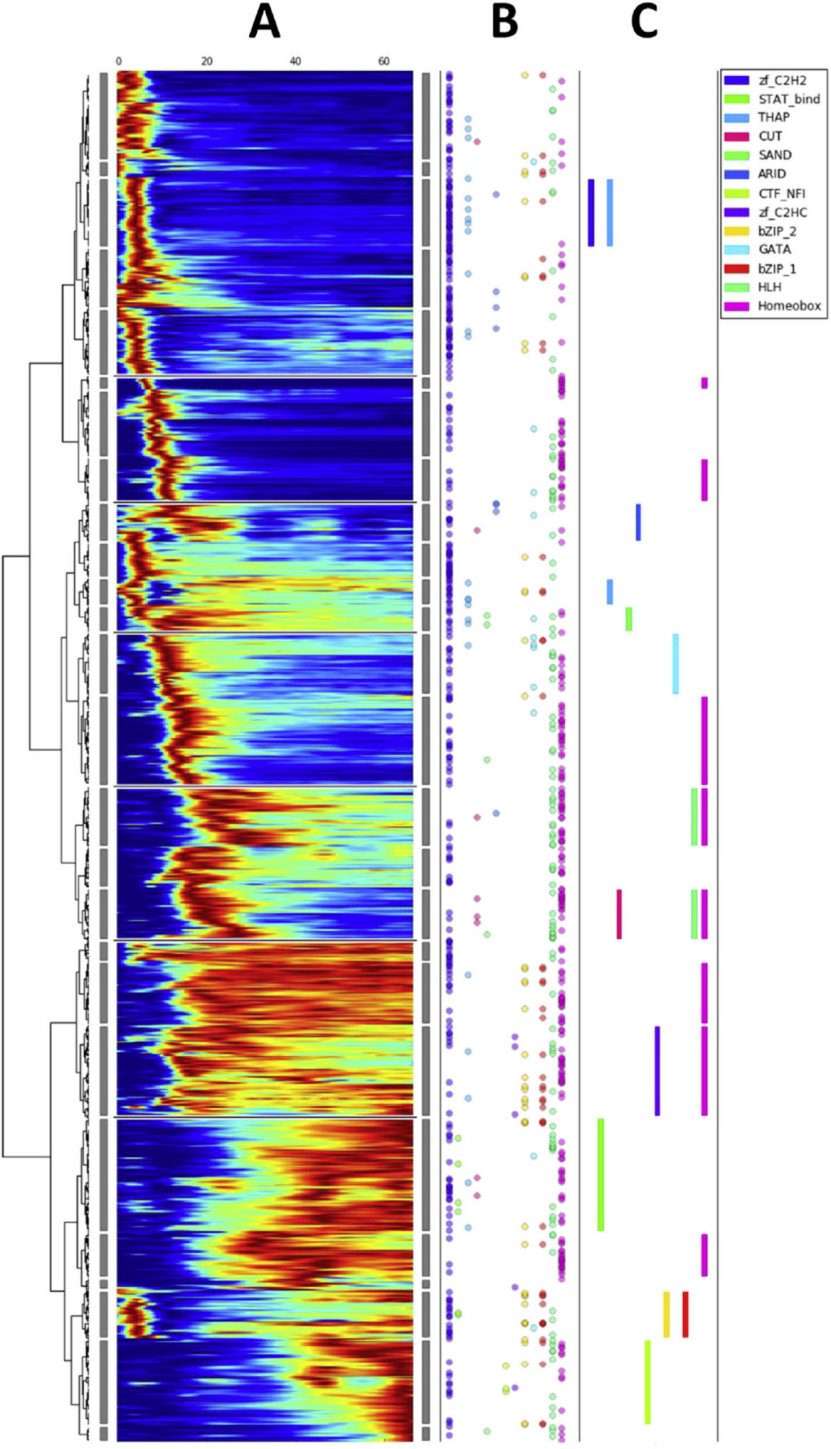
Transcription factor expression dynamics during early development. A. Heat-map of TF expression over the first 66 hours post fertilization. TFs were clustered on expression profiles and ordered according their average expression time. Vertical gray rectangles identify clusters. B. DBD families present in each TF are depicted as colored circles (only for those with significant family enrichment are shown) along the same horizontal lines as the expression profiles of the corresponding TFs. C. DBD family enrichment clusters (statistically significant with FDR < 0.1) are represented as rectangles.

We examined the global temporal expression dynamics of *X. tropicalis* TFs using the previously published high-resolution RNA-seq dataset, which covers early development from egg to tadpole and roughly corresponds to the first 8 weeks of human gestation (Owens et al., 2016). A clustered heatmap (Fig. 1) displays the expression patterns of TFs. Two main conclusions can be drawn from these observations. First, we find that the majority of TFs are expressed in “bursts” during relatively narrow time windows in embryogenesis. The heatmap shows that the timing of these bursts is not the same for all TFs. A significant minority of TFs have more complex patterns or a more sustained level of expression. Interestingly, there are few TFs that have a constant level of expression across the entire time-course, following zygotic genome activation. A second observation comes from interrogation of the data to determine whether TFs within specific DBD families are particularly enriched for expression during specific periods of development. Analysing groups of genes at significant cut-off levels in the dendrogram, we determined that TFs in 13 DBD families were statistically over-represented during the time intervals shown in the figure. For example, both zf_C2H2 and THAP family TFs are preferentially expressed in a burst of TF expression corresponding to genes activated at the midblastula transition.

### 3.4. Discovery of new TFs regulating early patterning of the primary germ layers

To understand better the spatial organization of TF usage in the early gastrula stage *Xenopus* embryo we screened for TFs with localized patterns of expression. The major onset of zygotic transcription occurs close to the mid-blastula transition with a smaller fraction of genes activated at earlier stages (Newport and Kirschner, 1987; Kimelman et al., 1987; Skirkanich et al., 2011; Yang et al., 2002; Paranjpe et al., 2013; Tan et al., 2013; Collart et al., 2014; Owens et al., 2016). Localized maternal determinants (e.g., mRNA encoding the Vegt and Foxi2 TFs) in the unfertilized egg establish early spatial asymmetries to set up the germ layers along the radially symmetrical animal-vegetal axis. Sperm entry breaks this symmetry to specify the dorsal-ventral axis and leads to the formation of Spemann's organizer in the dorsal equator. Numerous TFs are expressed regionally in response to these upstream maternal and dorsal-ventral cues (e.g., *sia1/2, gsc, ventx1/2, sox17*). We wished to identify all the TFs expressed in the early gastrula, and to determine which of these show localized spatial expression and are therefore likely to function in gene regulatory “subnetworks” that pattern the tissues of the early embryo.

**Fig. 2:**
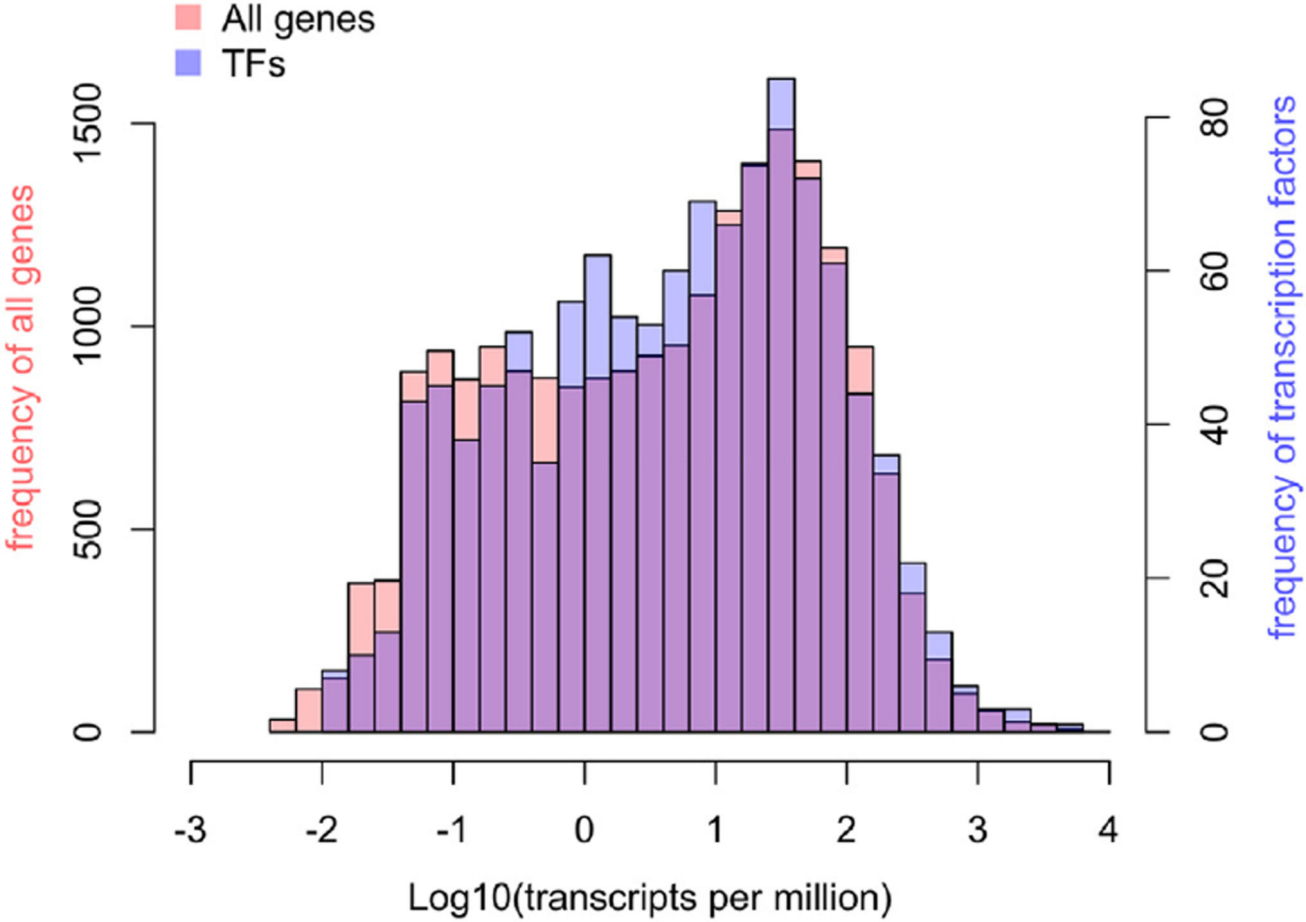
Distribution of TF gene expression levels in the early gastrula is indistinguishable from expression of all genes. The frequency of appearance of transcription factors (right vertical axis) and all genes (left vertical axis) is plotted as a function of expression level. The two distributions are nearly identical.

We first analyzed the expression of TFs in the whole early gastrula embryo RNA-seq data. We found that, at the sequencing depth used (replicates had 28 and 38M reads), 1130 TFs (91% of the catalog) were detectable at *any* expression level (Fig. 2; Supplementary Table 4). 785 TFs are expressed at or above 1 transcripts-per-million (TPM; Wagner et al., 2012) value, while 487 are expressed above a TPM of 10. Only 8 genes are expressed at a TPM value of 1000 or higher and these are *hmgb2, hmbgb3, ybx1, pou5f3.2, mixer, vegt, pou5f3.3* and *sp5l*. Interestingly, the distribution (Fig. 2) of TF expression levels is very similar to the distribution over all (21,056) expressed genes. This demonstrates that TFs, as a general category of genes, are not generally expressed at low levels relative to other genes, at least at the mRNA level.

**Fig. 3:**
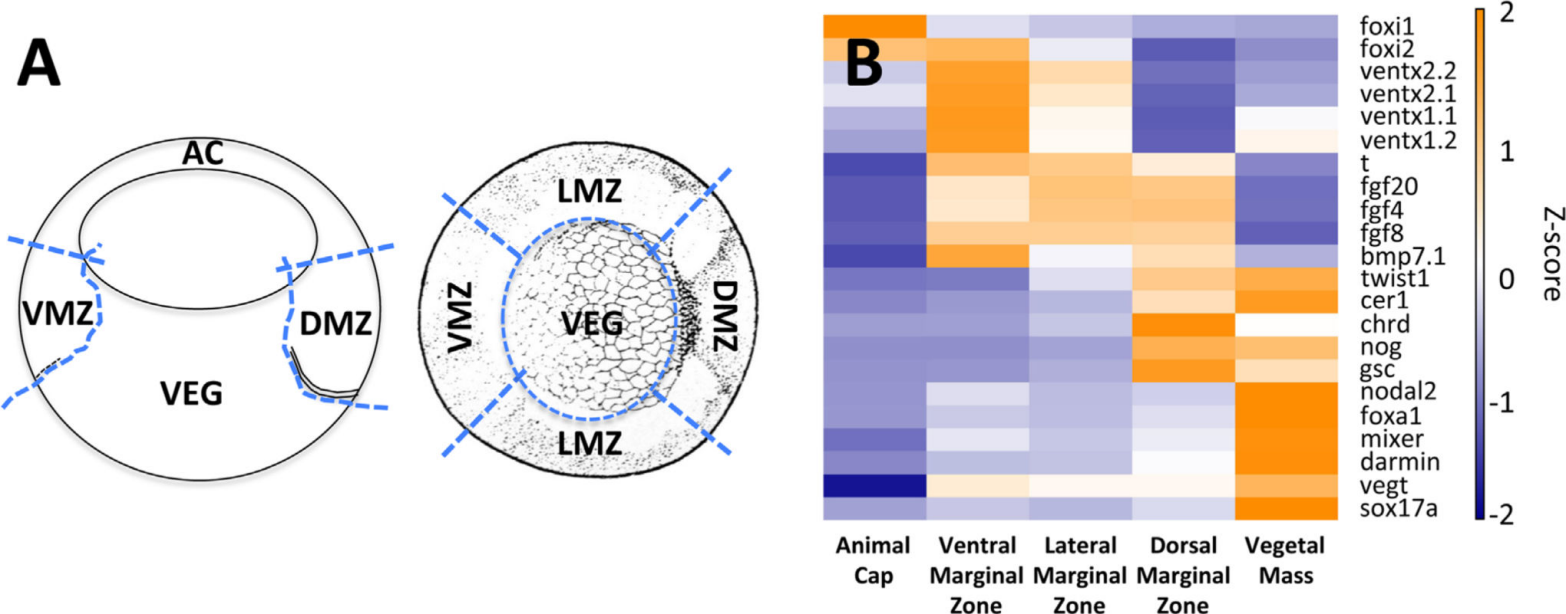
Gastrula dissection strategy and validation by marker gene expression in RNA-seq data. A. Illustration shows the locations of eyebrow hair knife cuts (dotted lines) for early gastrula dissections. Left shows a sagittal view while right shows a vegetal view. Dorsal is to the right. B. A heat map from RNA-seq data shows the relative expression of various marker genes in the different dissected embryo fragments. Abbr. AC, animal cap; DMZ, dorsal marginal zone; LMZ, lateral marginal zone; VMZ, ventral marginal zone; VEG, vegetal mass.

To perform a screen for TFs with spatially localized mRNA expression we dissected early gastrula embryos into 5 regions: animal cap (ectodermally enriched), vegetal mass (endodermally enriched), and dorsal, lateral and ventral marginal zones (mesodermally enriched) (Fig. 3A). We performed RNA-seq on mRNAs isolated from these regions, and on whole embryo mRNA collected from stage-matched sibling embryos. The entire experimental regimen was performed twice, using different clutches, to obtain biologically independent datasets. See Section 2 for more detail.

To confirm the quality of our dissections we examined expression of genes known to mark specific regions of the embryo (Fig. 3B). Genes expressed in the endoderm, including *sox17a, darmin, mixer, foxa1* and *nodal2*, had enriched expression in the vegetal mass dataset. High expression levels of *gsc, chrd* and *nog*, markers for Spemann organizer, were found in the dissected dorsal marginal zone, with weaker enrichment in the vegetal mass. Expression of the anterior endodermal marker *cer1* was confirmed in both dorsal marginal zone and more highly in the vegetal mass. The expression of mesodermal marker genes *t/brachyury, fgf4, 8* and *20* was found enriched in all marginal zone regions, with *t/brachyury* at a somewhat reduced level in the dorsal marginal zone consistent with its known expression pattern. Both *ventx1* and *2* expression was enriched in the ventral zone with reduced expression more laterally (ventral and lateral mesoderm) and animally (ectoderm), while *foxi1* and *2* were enriched in both the animal pole and ventral marginal zone regions. We conclude that the dissection faithfully captures known expression domains, with possible low level cross contamination across tissue boundaries.

**Fig. 4:**
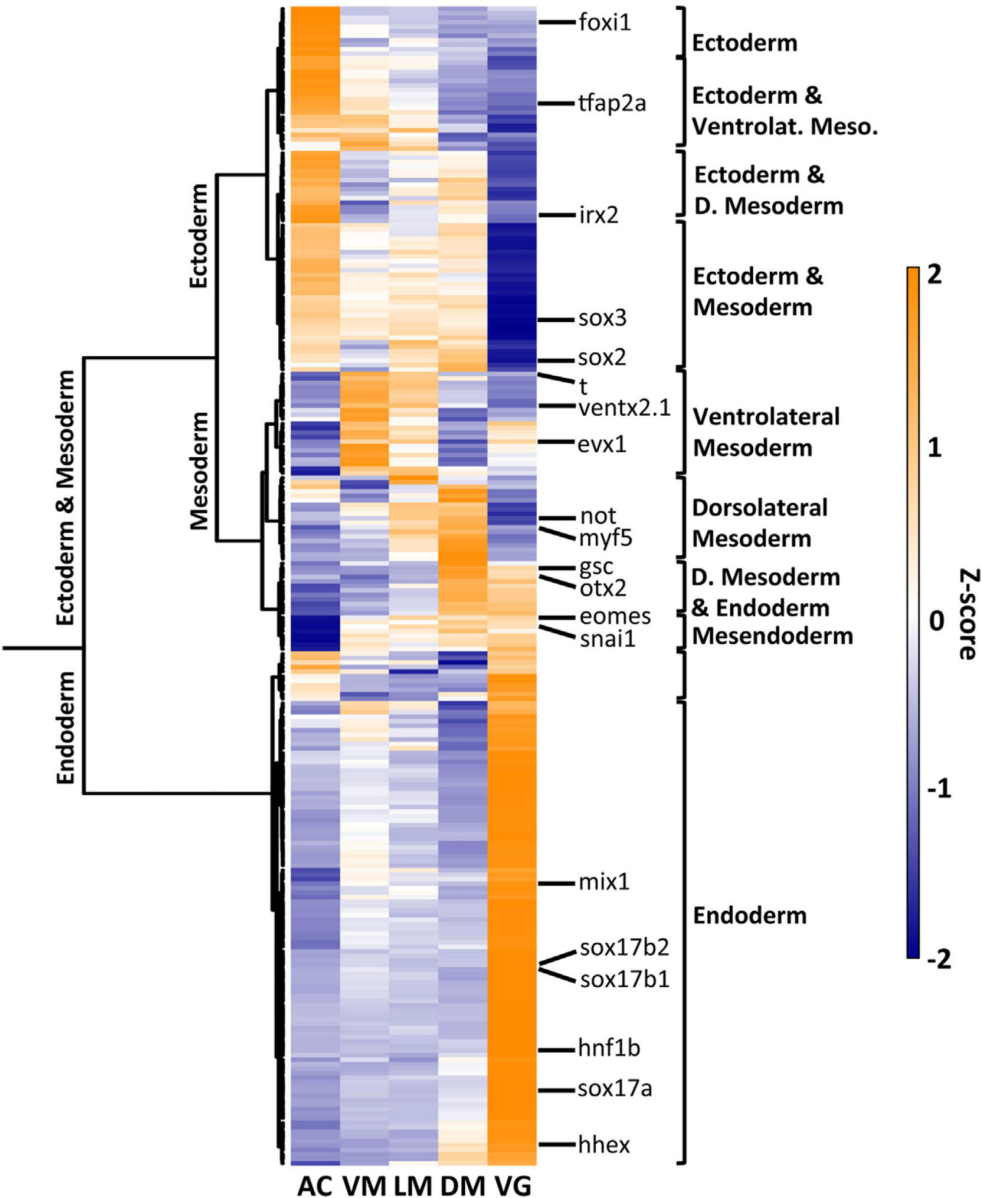
Spatial localizations of transcription factor RNAs derived from RNA-seq. A heatmap is shown to depict the spatial expression of differentially expressed TFs in the early gastrula. TF differential expression was determined between any comparison of two embryo fragments (posterior probability ≥ 0.95). The expression values are plotted as the z-score of each gene across embryo fragments. The labels (e.g. endoderm, ventral mesoderm) are the inferred predominant expression pattern in each major branch in the dendrogram.

We next interrogated the RNA-seq datasets for spatially localized expression of TFs. We performed a pairwise comparison between any two regions of the embryo and found 257 TFs with differential expression (Bayesian posterior probability statistical cutoff ≥ 0.95), and this was plotted as a clustered heatmap (Fig. 4). This shows major groupings of genes that we interpret as *predominantly* representing the three germ layers. We then compared dorsal to ventral marginal zones and also animal to vegetal expression values, applying stricter criteria: we enforced cutoffs of a minimum 2-fold expression ratio and a lower bound expression level of 1 TPM in the embryonic region of interest. TFs enriched in each of the four “poles”: dorsally, ventrally, animally, and vegetally, along with their TPM values, can be found in tables within Supplementary Table 5. We identify 30 TFs enriched dorsally (Spemann organizer region), 26 ventrally, 70 animally, and 130 vegetally. Using these criteria, the total number showing spatial enrichment along at least one axis is 218 TFs (38 TFs are differentially expressed along more than one axis).

While many of these differentially expressed TFs have previously characterized roles in early development, we found many that have not yet been reported in the early gastrula stage embryo, making these good candidates for further study. Most notable among these include *sebox* and *mxtx1*, which are strongly expressed in both vegetal endoderm and throughout the marginal zone (but not animally), and therefore may be involved in mesendoderm specification. *X. laevis sebox* is expressed in the mesoderm (Chen et al., 2015), but has not been reported in the vegetal endoderm, which is notorious for signal underrepresentation in in situ hybridization experiments. Zebrafish *mxtx1* and *2* are expressed in the extra-embryonic yolk syncytial layer (Hirata et al., 2000) but this gene has not been reported in *Xenopus* until the current study. Two genes expressed strongly in vegetal mass, while weakly or not expressed in the marginal zone and animal pole, are *uncx* and *gpbp1*, and are therefore good candidates for involvement in specification of endoderm. Two genes strongly expressed both animally and in the marginal zone are *foxh1.2* and *tead2* (Xetrov90019254, formerly *tead4*), suggesting roles for these in the ectoderm and mesoderm. A gene that is strongly expressed both dorsally and vegetally is *dmbx1*, which may play a role in Spemann's organizer. While *dmbx1* is known for its expression in the brain from work in a number of organisms, this TF has not yet been studied in the context of these other expression domains. However, amniote *dmbx1* was noted to be expressed in foregut endoderm at later stages of development (Gogoi et al., 2002) and therefore the expression we report in *Xenopus* gastrulae is consistent with involvement in the earliest stages of regional specification of the gut. Three genes found expressed both ventrally and vegetally are *tbx2, 3* and *cdx1*. All three are BMP responsive (Blitz and Cho, unpublished data) and based on this pattern are likely to play an early role in patterning of the posterior mesendoderm.

Our knowledge of the GRNs controlling germ layer specification and patterning is still quite primitive, as currently only tens of TFs have been studied in any detail, with only a few direct target genes identified in most cases. Thus, the GRNs of early development, and the functions of the TFs controlling them, remain largely unexplored. To stimulate progress towards a better understanding of the GRNs controlling early vertebrate development, we have here created a comprehensive catalog of the DNA-binding TFs in the genome of *X. tropicalis*. We find that *X. tropicalis* contains nearly all the TFs present in the mammalian genome, with a small number of these having undergone duplication since divergence from the last common ancestor with the mammalian lineage. This collection of TF information will be a useful resource for the *Xenopus* community, and contribute to a clearer picture of the gene repertoire of this important model system.

## Supplementary Material

1

2

3

4

5

6
